# Severe Erosive Esophagitis Developing After Gastric Ulcer Formation

**DOI:** 10.4021/jocmr895w

**Published:** 2012-09-12

**Authors:** Takatsugu Yamamoto, Koichiro Abe, Hajime Anjiki, Taro Ishii, Yasushi Kuyama

**Affiliations:** a Department of Internal Medicine, Teikyo University School of Medicine, Tokyo, Japan

**Keywords:** Esophagitis, Peptic ulcer, Elderly

## Abstract

A 90-year-old woman visited to our institute due to postprandial obstructive sensation of the esophagus. She had suffered from ischemic heart disease and diabetes mellitus, and taken low-dose aspirin for prophylaxis. She also had a history of a large ulcer located on the upper gastric body at 81 years-old. Esophago-gastric junction was normal excepting mild hiatal hernia at that time. The esophagogastroduodenoscopy showed a lump of food at the lower esophagus with severe stricture and mucosal injury. Rabeprazole 20 mg per day was given, and both the inflammatory change and the symptoms improved after the prescription. A probable reason of the development is impaired gastroesophageal motility and acid regurgitation induced by gastric deformity caused after ulcer formation.

## Introduction

The prevalence of reflux esophagitis increases in association with westernized diet and advanced aging society in Japan. However, it remains uncertain how esophagitis develops [1]. Here we report on a case of esophagitis appearing after 9 years.

## Case Report

**Figure 1:**
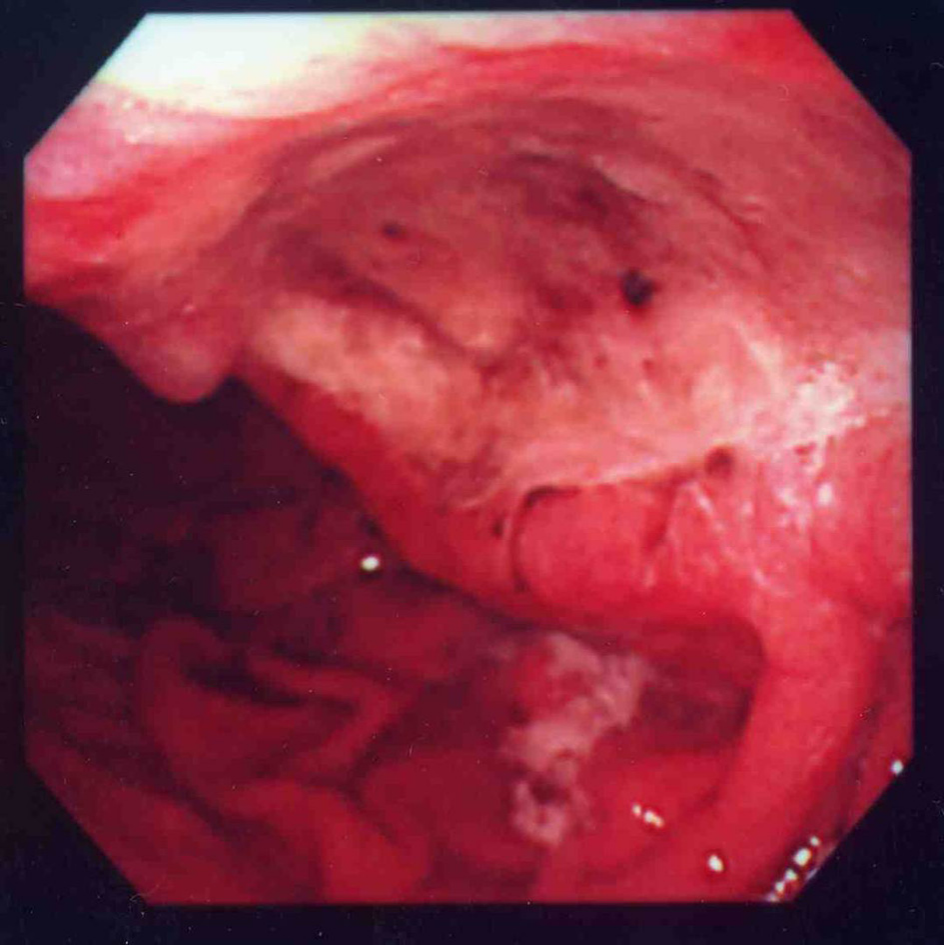
The patient had a large gastric ulcer when she was 81 years old.

**Figure 2:**
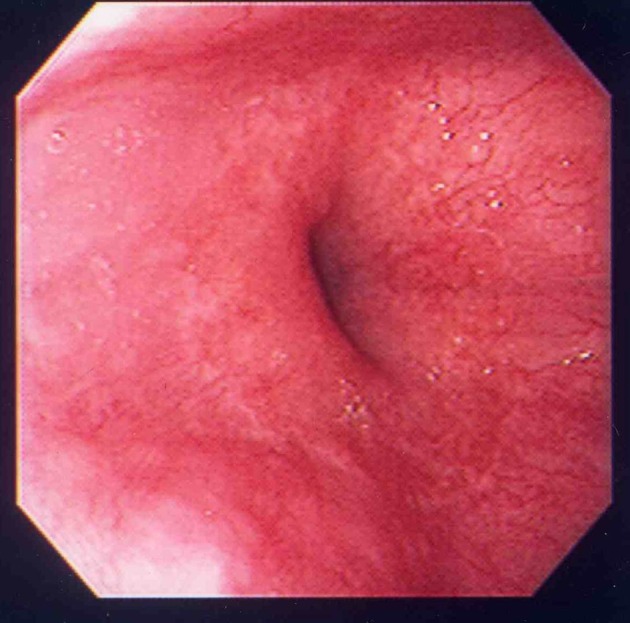
Slight hiatal hernia was seen at that time.

**Figure 3:**
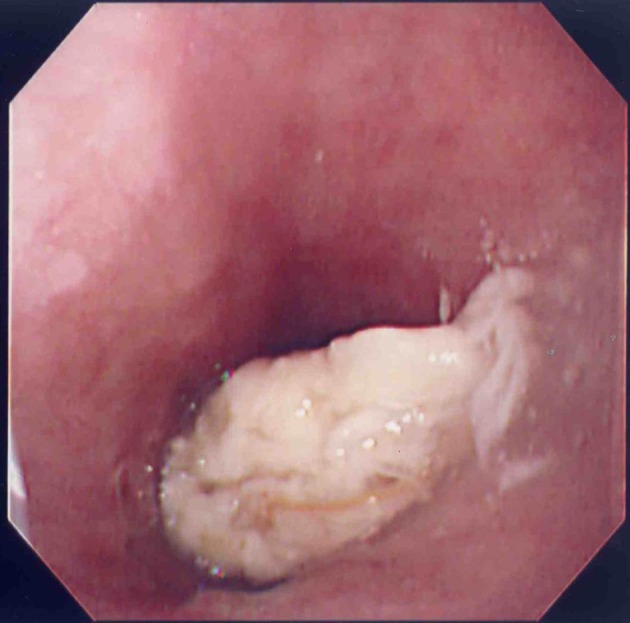
Esophagogastroduodenoscopy performed at age 90 showed a lump of food at the lower esophagus.

**Figure 4:**
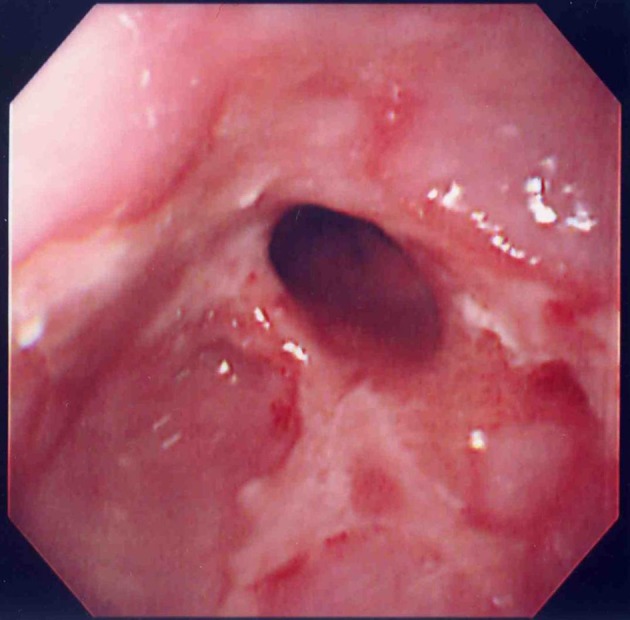
Esophageal stricture with severe mucosal injury was found after removal of food.

A 90-year-old woman visited to our institute due to postprandial obstructive sensation of the esophagus. She had suffered from ischemic heart disease and diabetes mellitus for over 30 years, and taken low-dose aspirin for prophylaxis. She also had a history of a large peptic ulcer located on the upper gastric body at 81-years old, and taken lansoprazole 15 mg per day since then (Fig. 1). Esophago-gastric junction was normal excepting mild hiatal hernia at that time (Fig. 2). The esophagogastroduodenoscopy showed a lump of food at the lower esophagus with severe stricture and mucosal injury (Fig. 3, 4). Gastric ulcer scar was also found without recurrence. Rabeprazole 20 mg per day was given, and both the inflammatory change and the symptoms improved after the prescription.

## Discussion

We described on the case with severe esophagitis developed 9 years after gastric ulcer formation. A probable reason of the disease is impaired gastroesophageal motility and acid regurgitation induced by gastric deformity. Because the course of developing esophagitis remains unknown, the present case gives us images that show how severe esophagitis develop.
